# Bulk and single-cell RNA sequencing data identified HMGB3 of chromatin regulators as a breast cancer biomarker associated with the cell cycle

**DOI:** 10.3389/fonc.2026.1835558

**Published:** 2026-06-11

**Authors:** Huan Wang, Luhui Li, Dingyuan Tu, Ping Shen, Zheyao Song, Mengjiao An, Xudong Li, Qi Ji, Peng Chen, Rongjun Cui

**Affiliations:** 1 Department of Biochemistry and Molecular Biology, Mudanjiang Medical University, Mudanjiang, Heilongjiang, China; 2 Department of Pathology and Transfusion Medicine, The 961st Hospital of the Joint Logistics, Support Force of The Chinese People’s Liberation Army, Qiqihar, Heilongjiang, China; 3 Department of Laboratory, Daqing Traditional Chinese Medicine Hospital, Daqing, Heilongjiang, China; 4 Department of Laboratory, Bei’an First People’s Hospital, Heihe, Heilongjiang, China; 5 Unit 93313, People’s Liberation Army of China, Changchun, China

**Keywords:** bioinformatics, breast cancer, chromatin regulators, immune microenvironment, single cell RNA sequencing technology

## Abstract

**Background:**

Chromatin regulators (CRs) have been reported to modulate tumorigenesis and progression in multiple pathophysiological processes, but little is known about theirs role and association with the immune microenvironment in breast cancer (BRCA).

**Methods:**

This study identified differential CRs gene expression in BRCA using transcriptome sequencing data and single-cell RNA sequencing data. We investigated the biological function of HMGB3 gene using western blot, transwell assay and immunohistochemistry (IHC).

**Results:**

In this research, eleven CRs were discovered and utilized to create a prognostic model. Using median risk scores, patients were sorted into high- and low-risk groups. The nomogram created from this model precisely predicted overall survival (OS). Low-risk patients had notably higher immune cells infiltrating. IHC provided additional evidence that the expression levels of HMGB3 are closely linked to BRCA. *In vitro* functional assays demonstrated that HMGB3 enhances the invasion of BRCA cells and facilitates malignant progression by controlling cell cycle.

**Conclusion:**

Based on bioinformatics analysis of the prognostic value of CRs in BRCA, we identified that HMGB3 as a novel target molecule to promote the occurrence and development of BRCA.

## 1 Introduction

Breast cancer (BRCA) is one of the most common cancers in women and the leading cause of cancer deaths worldwide (1). In 2022, global statistics show that there were 2.3 million new BRCA cases, making up 25% of all female cancer cases, and 670,000 deaths, which accounted for 15.5% of all female cancer fatalities (2). Although new treatments for BRCA, such as immune checkpoint (3) inhibitors and antibody-drug conjugates (4) have been introduced, BRCA remains prevalent. If the current trends continue, it is projected that by 2050 there will be 3.2 million new cases of BRCA and 1.1 million deaths related to BRCA, marking increases of 38% and 68% from the year 2022 (2). Therefore, the ongoing search for early biomarkers of BRCA remains particularly important.

Changes in epigenetics caused by chromatin regulators (CRs) are a recognized characteristic of cancer (5). CRs are a class of proteins possessing specialized functional domains that dynamically regulate chromatin structure. CRs are primarily categorized into three major classes: DNA methylation, histone modifications, and chromatin remodeling factors according to their roles in epigenetics (6, 7). They are closely intertwined in biological processes. Growing evidence suggests that abnormal expression of chromatin regulators is strongly linked to various biological processes such as inflammation and immune response (8, 9). Notably, disruption of CRs is commonly observed across various cancers, and its abnormal expression and mutations are significantly associated with the prognosis of malignant tumors (10, 11).

The tumor microenvironment (TME) comprises diverse immune cells and stromal cells, which play a crucial role in the progression of malignant tumors and treatment response (12). Tumor cells functionally remodel the surrounding microenvironment by secreting various cytokines, chemokines, and other factors, leading to the re-editing of the TME (13). In recent years, immunotherapy has represented a major breakthrough in cancer treatment, with multiple cancer types demonstrating sustained clinical responses to immunotherapy (14).

Single-cell RNA sequencing (ScRNA-seq) enables in-depth analysis of the transcriptional states of thousands of individual cells. Its emergence has fundamentally overcome the limitations of traditional transcriptomic sequencing for gene detection, allowing for higher-resolution mapping of the entire transcriptome and distinguishing different cell types within tumor tissues. Currently, scRNA-seq technology has facilitated the classification of many malignant tumors based on cell types, including pancreatic cancer and clear cell renal cell carcinoma (15). Moreover, scRNA-seq holds promise for expanding clinical applications in refractory cancer cases and emerging as a non-invasive method for detecting circulating tumor cells, analyzing intratumoral heterogeneity, and sensitively estimating tumor recurrence (16).

As far as we know, there have been no studies examining the combined effects of CRs and the TME landscape using scRNA-seq for prognostic prediction in BRCA. In this analysis, the prognostic model we created using risk scores accurately distinguished between high-risk and low-risk patient groups. Differences in tumor mutation burden, immune-related genes also unveiled the value of the classifier. Further clinical sample verification and *in vitro* experiments demonstrated that the high expression of HMGB3 in BRCA tissues, through the cell cycle pathway, HMGB3 facilitates the proliferation and invasion of BRCA cells.

## 2 Materials and methods

### 2.1 Data collection and identification of differentially expressed CRs

Transcriptomic and clinical data for BRCA samples were extracted from The Cancer Genome Atlas Program (TCGA), a total of 870 CRs were retrieved from a previous study (5). Using the relevant ‘R’ package, these mRNA expression profiles were normalized. To identify differentially expressed genes (DEGs), differential expression analysis was carried out using the ‘limma’ package, with criteria of |logFC|> 0.4 and an adjusted p-value < 0.05. Heatmaps were used to present differences of these CRs in BRCA.

### 2.2 GO and KEGG

Gene Ontology (GO) and Kyoto Encyclopedia of Genes and Genomes (KEGG) analyses were performed to explore the molecular mechanisms underlying differentially expressed CRs with ‘clusterProfiler’, ‘org.Hs.eg.db’ and ‘enrichplot’. *P* value < 0.05 and FDR < 25% were considered statistically significant. The enrichment results were then visualized using the ‘ggplot2’ package.

### 2.3 Construction and validation of a prognostic model based on CRs

A univariate Cox regression analysis was performed by us to further examine the prognostic implications of CRs. Following that, the lasso Cox regression model was used to find genes that are closely related. Ultimately, the prognostic risk model was developed using multivariate Cox regression analysis with the ‘glmnet’ R package. Risk scores were calculated by the following tool: RiskScore= $\sum_{i=1}^{n}\beta i\times expression\;of\;mRNA$ where Coef is multivariate Cox regression model coefficient of the corresponding mRNA. Combine clinical pathology data and risk scores in R using the ‘intersect’ command. Kaplan-Meier (K-M) curves were utilized to conduct survival analysis and assess the prognosis of the two groups. To evaluate the prognostic potential of the risk model, time-related ROC analysis was conducted using the survival ROC package.

### 2.4 Exploration of the clinical and predictive value of the prognostic risk model

Age, S stage, T stage, N stage, M stage, and risk score were part of the univariate and multivariate Cox regression analysis to find independent risk factors related to prognosis. To enhance prognosis prediction, we employed the ‘regplot’ package to build a nomogram capable of estimating survival for 1, 3, and 5 years. Time-dependent C-index and ROC curve analysis were employed to determine the model’s accuracy. Finally, calibration plot was generated using the ‘rms’ package.

### 2.5 Analysis of tumor mutational burden and tumor immune microenvironment

Immune infiltration results from tumor samples of 1,105 BRCA patients were acquired through TIMER2.0 (http://timer.cistrome.org/), seven database-based computational methods—TIMER, XCELL, QUANTISEQ, MCPCOUNTER, EPIC, CIBERSORT-ABS, and CIBERSORT—were employed to assess the relationship between high-risk and low-risk groups and immune-infiltrating cells. Calculate the relative proportions of 22 immune cell types in high-risk and low-risk groups through the CIBERSORT. BRCA patients were divided into groups with high and low immune scores based on immune function scores for survival analysis. GSVA package evaluated the correlation between 13 immune function genes and BRCA transcriptome expression. The Wilcoxon signed-rank test was employed to identify differences between the high-risk and low-risk groups in terms of immune-infiltrating cells and immune-related functional pathways.

### 2.6 Parallel analysis of scRNA-seq data

A dataset comprising scRNA-seq of BRCA (GSE180286) was obtained from the Gene Expression Omnibus (GEO). Begin by eliminating the cells of poor quality. Using the ‘LogNormalize’ method, the data was standardized to find the 1,500 genes with the most significant intercellular coefficient of variation. Principal component analysis (PCA) is utilized to decrease the number of dimensions in gene data. To further cluster the characteristic genes, the t-Distributed Stochastic Neighbor Embedding (T-SNE) algorithm was utilized. Using the ‘monocle’ package for analyzing cell states and their pseudo-time trajectories. The R ‘ggplot2’ package was used for analyses of differential CRs expression.

### 2.7 Cell line culture

The human BRCA cell lines (MDA-MB-231) were procured from the cell bank of Saibai Kang (Shanghai) Biotechnology Co. Ltd. All cells were cultured in complete medium with 10% fetal bovine serum and1% penicillin-streptomycin (FBS, Gibco brand), under incubator conditions of 5% CO2, 37°C, and 95% humidity.

### 2.8 Immunohistochemical staining for protein expression in tumor tissue

Both tumor and normal hydrated sections were obtained from Department of Pathology, 961 Hospital, Joint Logistics Support Force, subsequently, the sections were subjected to antigen retrieval by boiling in buffer for 25min, incubated in 3% H2O2 at room temperature for 10min to block endogenous peroxidases, and then treated with 5% BSA solution for 30min at 37°C. And the sections were incubated with primary HMGB3 (1:50) antibody at 4°C 12h, followed by PBS washing and incubation with secondary IgG(H+L)(1:10) antibody for 30min at 37°C. Following another round of PBS washing, DAB was used for color development for 3–5min. Counterstaining with hematoxylin was performed for 3min, followed by differentiation in hydrochloric acid alcohol.

### 2.9 Transwell assay

Transwell assays were conducted for cell invasion evaluation. Prior to the experiment, treated cells were placed in the upper chamber with serum-free medium, adjust the density to 5 × 10^5^ cells/mL. The lower chamber contained 500µL∼600µL of 10% PBS medium. Fix with 4% paraformaldehyde for 60 minutes, then stain with 0.1% crystal violet for 1 hour. After staining, use a cotton swab to remove the inner chamber cells. Invert the chamber onto a microscope slide for photography. Absorbance was then measured using a spectrophotometer.

### 2.10 Western blot analysis

Using RIPA (TTermo Fisher Scientiffc #89,900) lysis buffer, cells were extracted at a temperature of 4°C. Cell lysates were centrifuged at 12,000 rpm for 10 min, and the supernatants were collected and analyzed for protein concentration using a PierceBCA protein assay kit (TTermo Fisher Scientiffc #23225). Run SDS-PAGE for 1.5 hours and then proceed with a 1-hour transfer to a membrane. After sealing the PVDF membrane with skim milk powder, incubate the primary antibody overnight at 4°C. Following membrane washing, incubate the secondary antibody for 2 hours. Moisten the PVDF membrane with a highly sensitive chemiluminescent solution and develop using an imaging system.

### 2.11 Statistical analysis

GraphPad Prism 9.0 software is used for graphing and statistical analysis. Each experiment was performed in triplicate biological repeats (n=3). One-way ANOVA is used for quantitative comparisons between multiple groups. The S-N-K method is used for pairwise comparisons. Survival analysis was performed using the K-M method, and comparisons were made using the log-rank test. The Wilcoxon signed-rank test was employed to analyze differences in risk scores based on immune-infiltrating cell content significance was set at *p* < 0.05.

## 3 Results

### 3.1 Identification of differentially expressed CRs in breast cancer samples

**Supplementary Figure 1** shows the expression of differentially expressed CRs. Differential expression analysis of 1105 BRCA tumor samples and 112 para-tumoral breast samples from TCGA, identifying 136 dysregulated CRs in BRCA, with 57 downregulated and 79 upregulated (|logFC| > 0.4, *p* < 0.05).

### 3.2 Enrichment analysis of GO and KEGG

**Figure 1:**
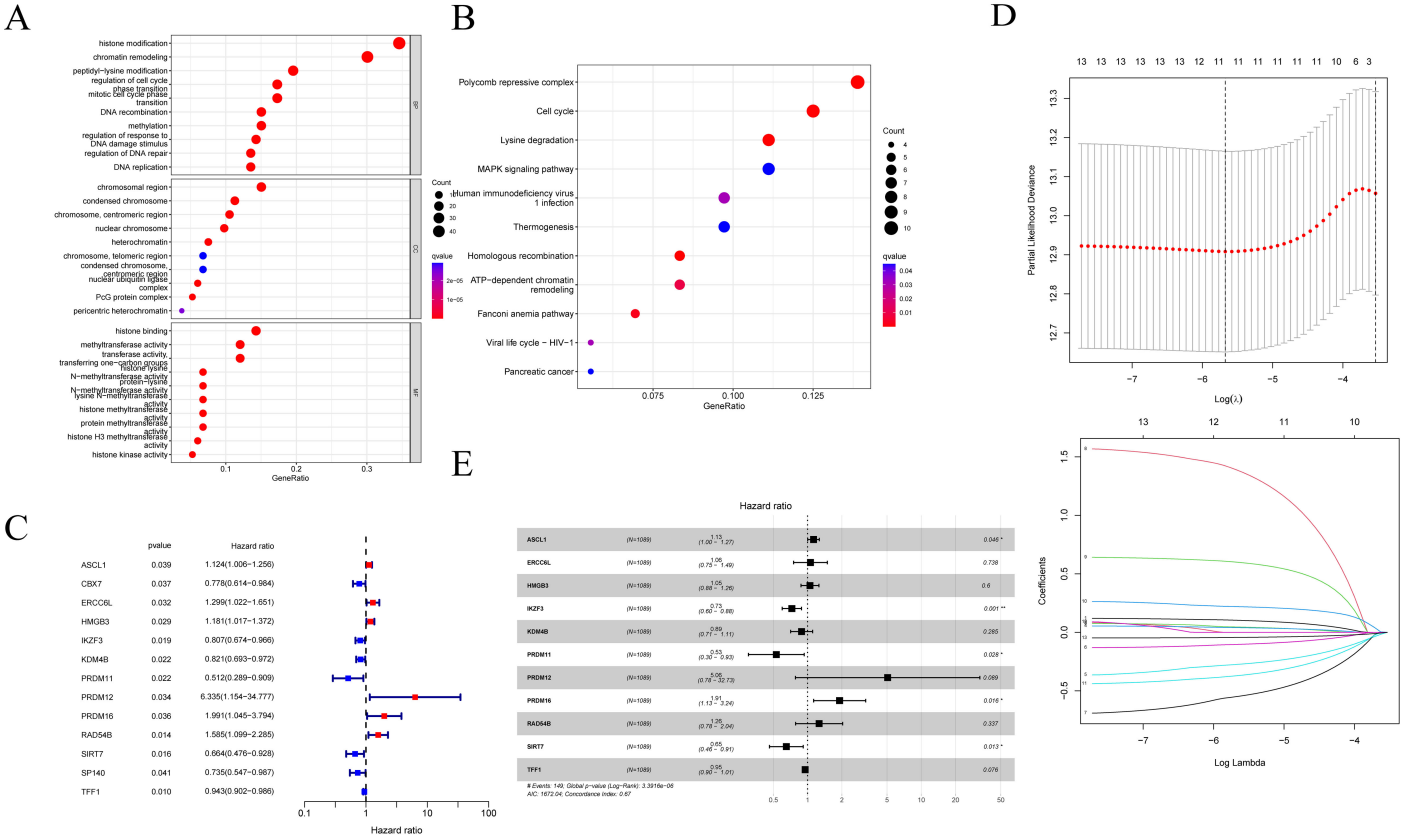
Identification and prognostic model construction of BRCA related CRs. **(A, B)** The bubble plot displays significant of GO and KEGG enrichment results. (Count: The count of genes that are notably enriched in this pathway; Color: Significant pathway enrichment is shown by the logarithm of the p-value. A deeper red color indicates a more significant p-value.) **(C, E)** Cox regression analysis. **(D)** LASSO coefficient curves.

Using DAVID, GO and KEGG enrichment analyses were carried out to explore the biological functions and signaling pathways linked to differentially expressed CRs. The GO enrichment analysis (ranked by count) (**Figure 1A**) revealed that these CRs were primarily associated with histone modification and chromosome remodeling, molecular functions are mainly associated with histidine-rich calcium-binding proteins and methyltransferase activation. **Figure 1B** shows the top 11 KEGG pathways (ranked by count) involving differentially expressed CRs, which included polycomb repressive complex, cell cycle, lysine degradation, etc. The results from the annotations demonstrated a tight association between these genes and the emergence of tumors.

### 3.3 Construction of CRs prognostic model

To determine the prognostic value of CRs, we applied univariate Cox regression analysis on the deregulated CR. 13 CRs were recognized as factors in overall survival (**Figure 1C**). Afterward, the LASSO Cox regression model was utilized to detect genes with strong correlations to BRCA and to build the prognostic CRs (**Figure 1D**). In addition, multivariate Cox regression (**Figure 1E**) analysis was used to further verify the results and correlation coefficients were obtained using the formula: Risk score = (0.12 × expression of ASCL1) + (0.056 × expression of ERCC6L) + (0.047 × expression of HMGB3) + (-0.319 × expression of IKZF3) + (0.121 × expression of KDM4B) + (-0.628 × expression of PRDM11) + (1.645 × expression of PRDM12) + (0.639 × expression of PRDM16) + (0.241 × expression of RAD54B) + (-0.432 × expression of SIRT7) + (-0.049 × expression of TFF1).

### 3.4 Prediction of the robustness of CRs prognostic model

**Figure 2:**
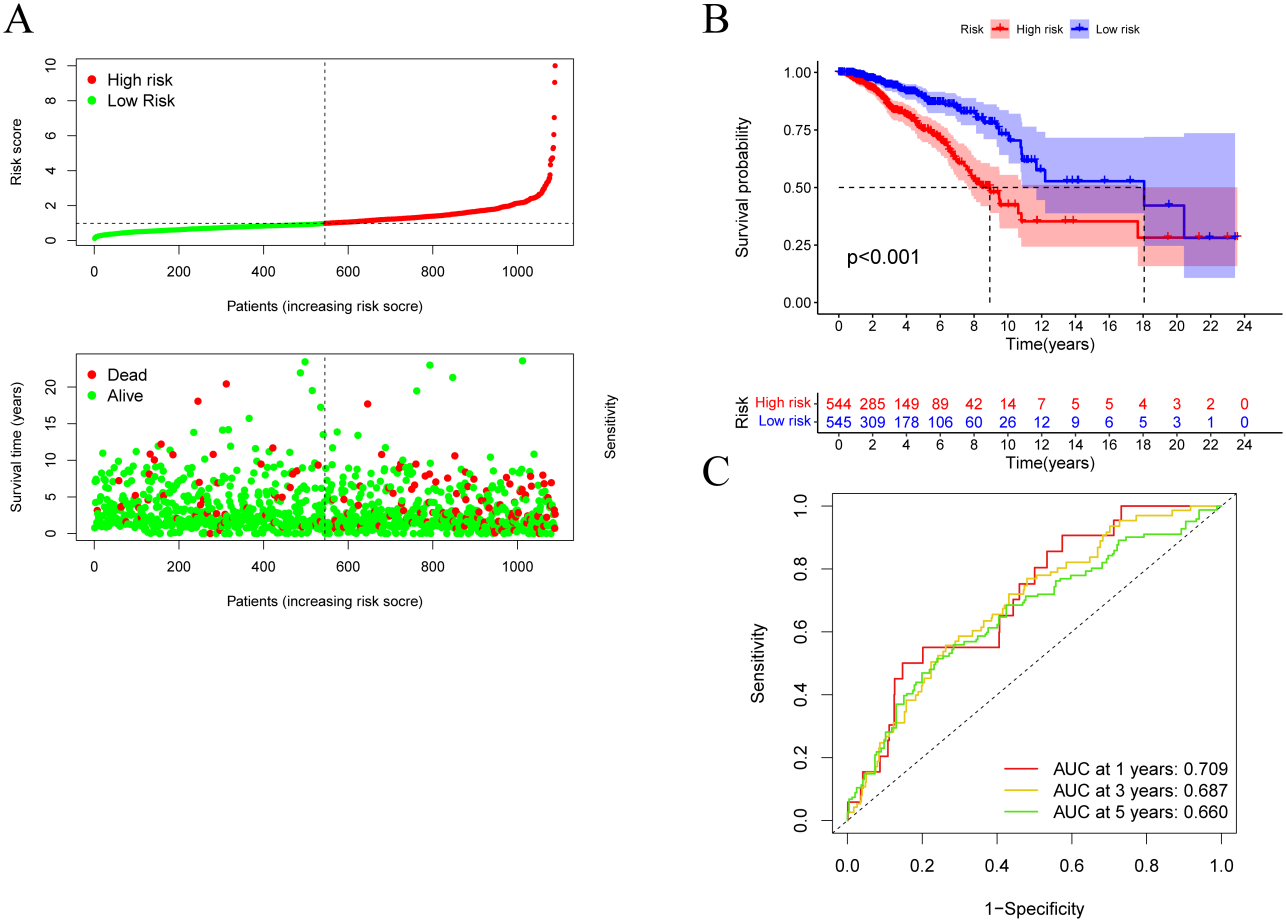
Survival analysis and validation **(A)** survival status graphs. **(B)** K-M curves. **(C)** Time-dependent ROC curves for overall survival.

BRCA patients were divided into high-risk and low-risk categories based on the median risk score. Deaths were significantly higher in the high-risk group than in the low-risk group (*p* < 0.001), which implies a negative correlation between risk score and prognosis (**Figure 2A**). K-M analysis revealed a significant connection between the risk score and overall survival (OS) in BRCA patients. The low-risk score group had a better prognostic outcome than the high-risk group (*p* < 0.001) (**Figure 2B**). ROC analysis demonstrated that the AUC for BRCA patients was 0.709 at 1 year, 0.687 at 3 years, and 0.660 at 5 years (**Figure 2C**), implying that this prognostic model holds predictive value for the prognosis of individuals with BRCA.

### 3.5 Evaluation of prognostic model’s clinicopathological features

**Figure 3:**
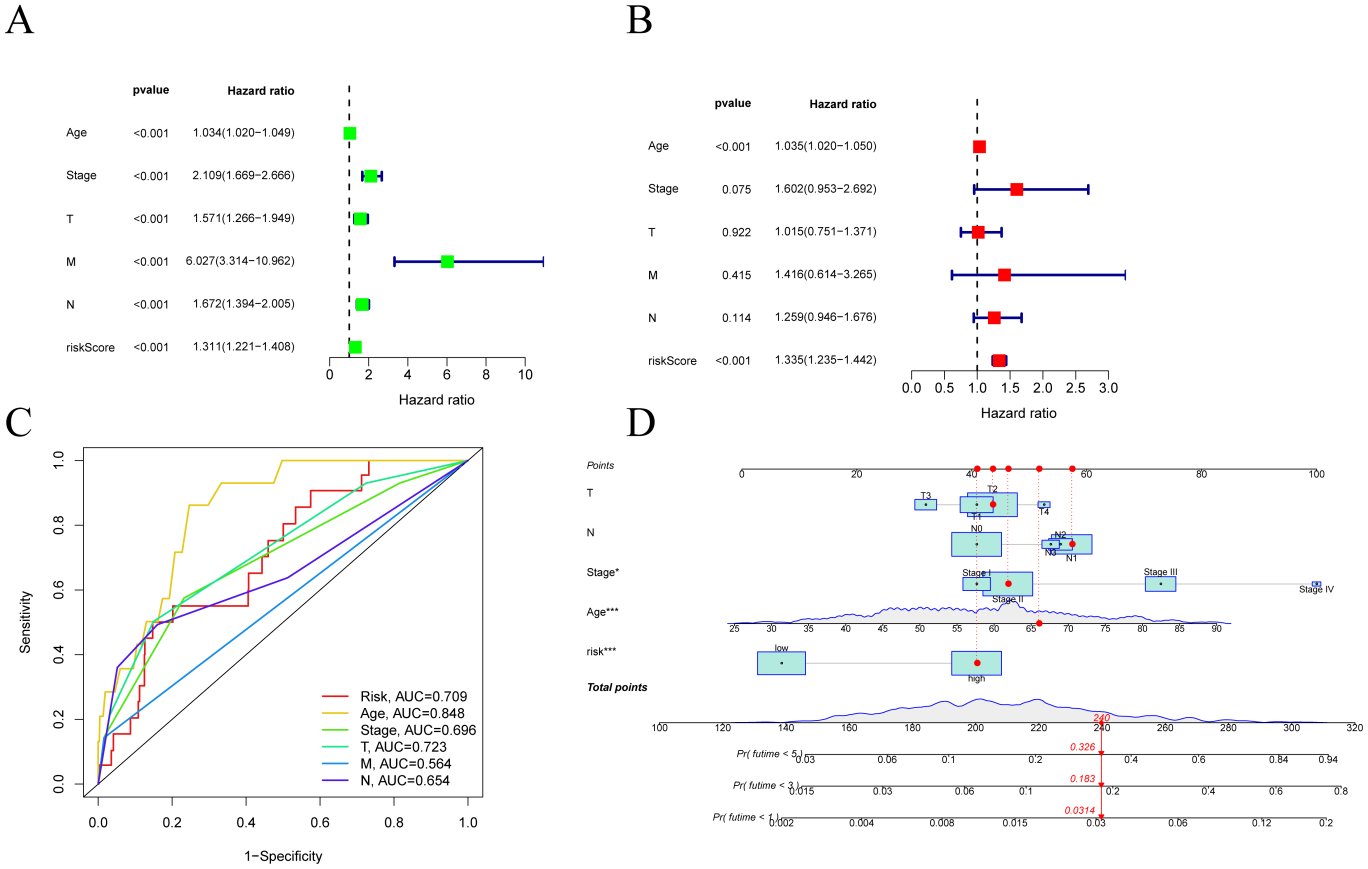
Clinical evaluation of predictive model risk scores. **(A, B)** Cox regression analysis. **(C)** ROC curves. **(D)** The nomogram was developed based on age and risk score.

We executed univariable and multivariable Cox analyses to testify whether this signature could be an independent prognostic indicator. Univariate analysis showed that risk score, S stage, N stage, T stage, and age were significantly relevant to the survival of BRCA patients (*p* < 0.001) (**Figure 3A**). Multivariate analysis indicated that the risk score and age were still remarkably related to prognosis (*p* < 0.05) (**Figure 3B**). ROC analysis showed that the AUC for risk score (0.709), which suggested that risk score had certain predictive ability in the prognosis of BRCA. These results demonstrated that CR-based signature was an independent prognostic indicator for BRCA patients (**Figure 3C**). A nomogram was developed by combining the risk score and the clinicopathologic staging to quantify the risk score and survival probability of BRCA patients (**Figure 3D**).

### 3.6 Evaluation of tumor-immune landscape and analysis of immune related function

To assess the differences in the immune microenvironment of BRCA patients with different risk scores, we utilized the immune microenvironment assessment tool, some online database of immune cell infiltration, immune checkpoint gene expression, and immune function scores of BRCA patients with different risk scores.

**Figure 4:**
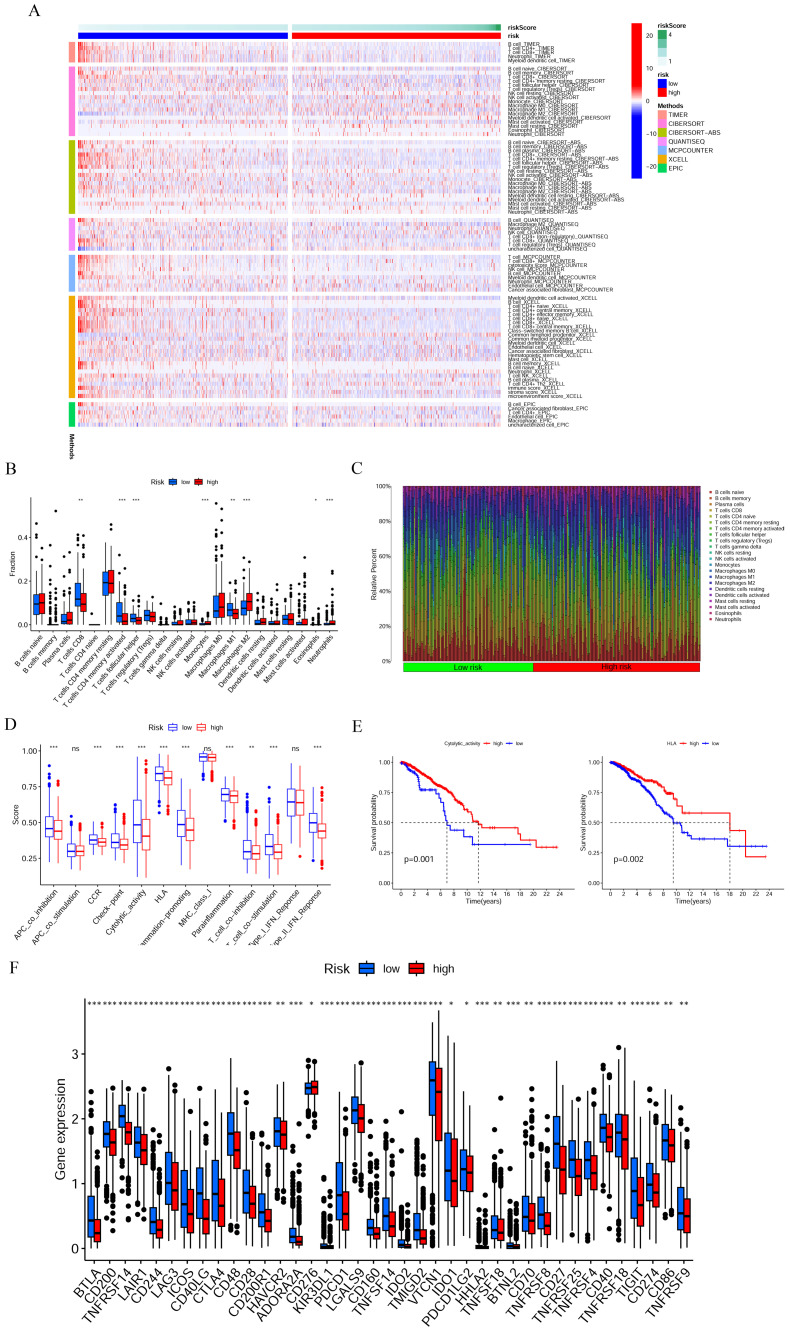
Immunological relevance analysis. **(A)** Stacked bar charts showing the composition ratios of classic immune cells in BRCA patient samples. **(B, C)** Immune cell infiltration in the high- and low-risk groups. **(D)** Immune functionality of BRCA patients by risk-score. **(E)** K-M curves of BRCA patients by immune function. **(F)** Expression of 22 immune checkpoint-related genes in tumors of BRCA patients grouped by risk-score.

Firstly, we found that patients in the low-risk group had significantly higher adaptive immunity scores than those in the high-risk group (**Figure 4A**), suggesting that stronger immune escape may occur in the high-risk group.

The CIBERSORT algorithm was used to demonstrate the differences in specific immune cell infiltration between high and low risk groups, and the results showed that the infiltration of CD8+ T cells, activated CD4+ memory T cells, follicular helper T cells, and macrophage M1 was higher in the low-risk group, whereas monocytes, and macrophage M2 were more infiltrated in the high-risk group (*p* < 0.05) (**Figure 4B**). The proportions of the 22 immune cells in samples from high and low risk groups are detailed in **Figure 4C**. In the high-risk group, GSVA analysis indicated a reduction in 10 of the 13 immune functions. Except for the coactivation of antigen-presenting cells, MHC class 1 molecules, and the type I interferon response, immune evasion was stronger (**Figure 4D**), and that a low immune response was linked to poorer survival prospects (**Figure 4E**). More precisely, low-risk BRCA patients exhibited higher scores for immune cell infiltration than high-risk patients in the majority of cell types, such as CD274, PDCD1, CTLA4, TIGIT, LAG3 (**Figure 4F**). A correlation analysis found that the group with high-risk scores had an increased tumor mutation burden compared to another (**Supplementary Figure 2**).

### 3.7 Concurrent analysis of data from scRNA-seq

**Figure 5:**
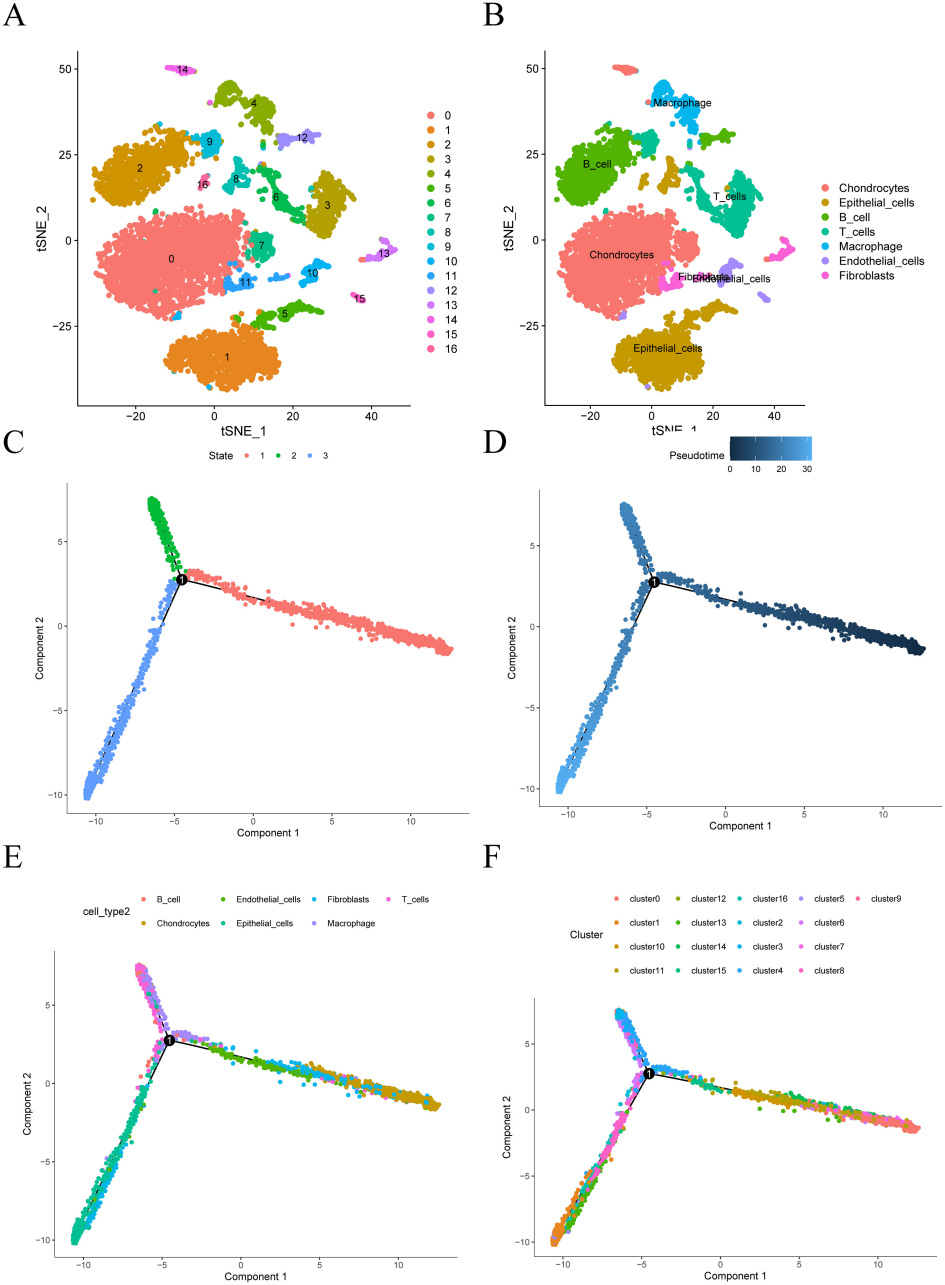
ScRNA-seq parallel analysis. **(A, B)** Cell annotation. **(C, D)** Analysis of cell state trajectories. **(E, F)** Analysis of cell differentiation using pseudo temporal trajectories.

We obtained 16,384 high-quality single-cell transcriptomes from 5 BRCA patients using the dataset GSE180286. Using the LogNormalize method, gene expression data were normalized, and 1500 highly variable genes were pinpointed with the ‘vst’ method (**Supplementary Figures 3A–D**). PCA facilitated dimensionality reduction, and the foremost 15 principal components were employed to correct batch effects (**Supplementary Figure 3E**). To achieve a more precise clustering of cell populations, we utilized the t-SNE algorithm, a common tool for high-dimensional data visualization, where we effectively divided the samples into 17 subcategories(**Figures 5A, B**). Reference datasets from the Human Primary Cell Atlas (HPA) are used to annotate cells in each cluster. Using the monocle ‘R’ package, we performed a pseudo chronological analysis on malignant cell clusters to reveal their transition from childhood to exhaustion (**Figures 5C, D**). The analysis indicated that the possible differentiation paths of the malignant cells included three distinct states. Interestingly, we observed that the initial cells were mainly fibroblasts, chondrocytes, and endothelial cells. Late-stage cells were mainly epithelial, with notable infiltration of T cells and macrophages (**Figures 5E, F**).

### 3.8 Combining bulk with scRNA-seq reveals important CRs.HMGB3 is linked to cell cycle

**Figure 6:**
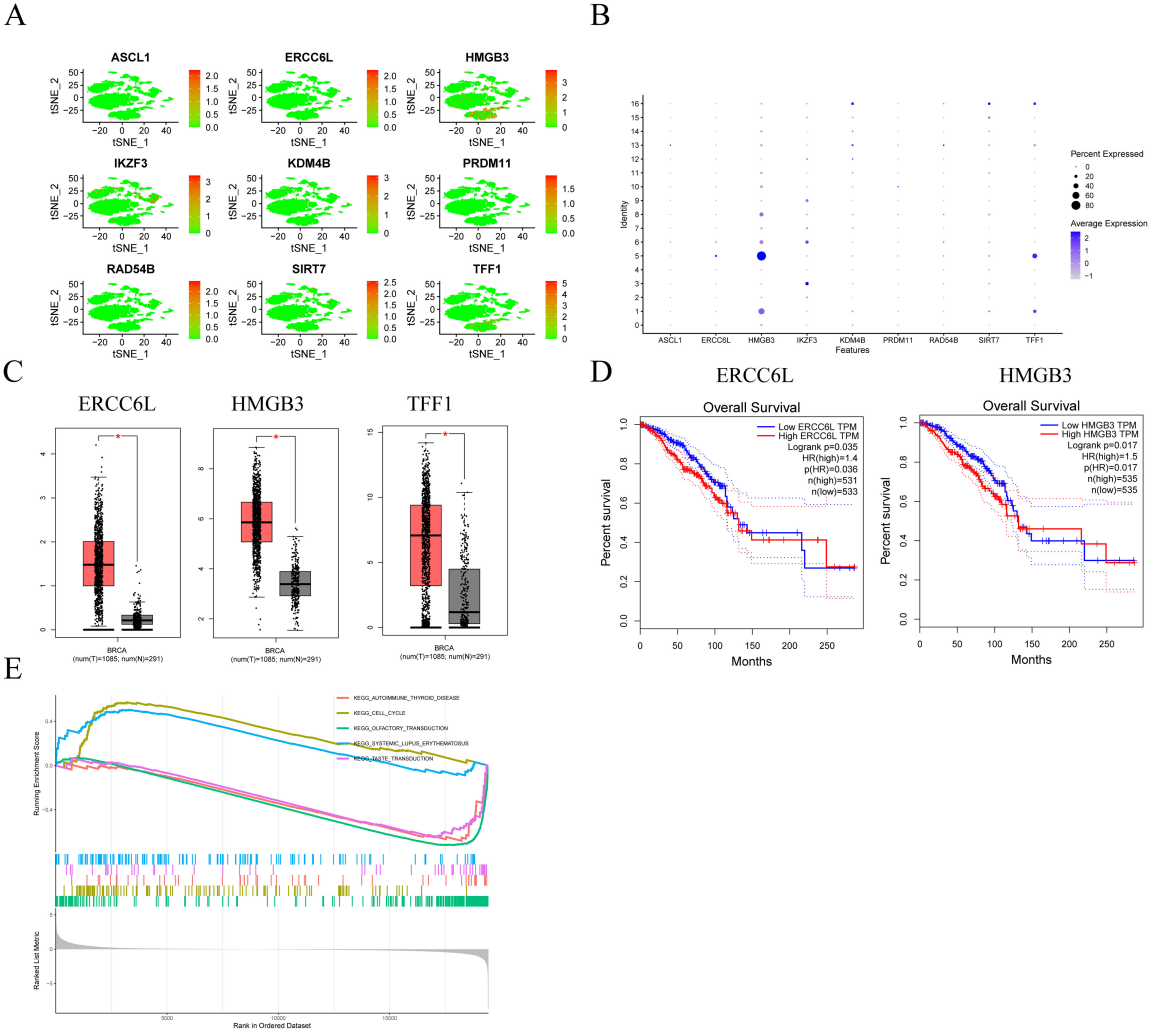
HMGB3 is linked to cell cycle **(A, B)** Expression of shared genes in each cell cluster. **(C, D)** Expression Differences and K-M curves in common genes in BRCA. **(E)** GSEA of HMGB3.

Nine co-expressed CRs (ASCL1, ERCC6L, HMGB3, IKZF3, KDM4B, PRDM11, RAD54B, SIRT7, TFF1) were identified from the intersection of 11 genes from CRs-related prognostic models and 4,965 marker genes from scRNA-seq data. The distribution of nine genes across different clusters is illustrated in **Figures 6A, B**, highlighting that HMGB3 and TFF1 are chiefly present in Cluster 5 (epithelial cells).

GEPIA data reveals that ERCC6L, HMGB3, and TFF1 are significantly more expressed in BRCA tissue than in nearby non-cancerous tissue (**Figure 6C**). The prognostic importance of ERCC6L and HMGB3 expression is notable in BRCA tissues (**Figure 6D**).

Based on GSEA analysis, HMGB3 overexpression is largely positively linked to the cell cycle signaling pathway, while it is negatively linked to olfactory and gustatory signal transduction (**Figure 6E**). This suggests that HMGB3 expression might be closely linked to the cell cycle in BRCA.

### 3.9 Expression analysis of HMGB3 in BRCA

**Figure 7:**
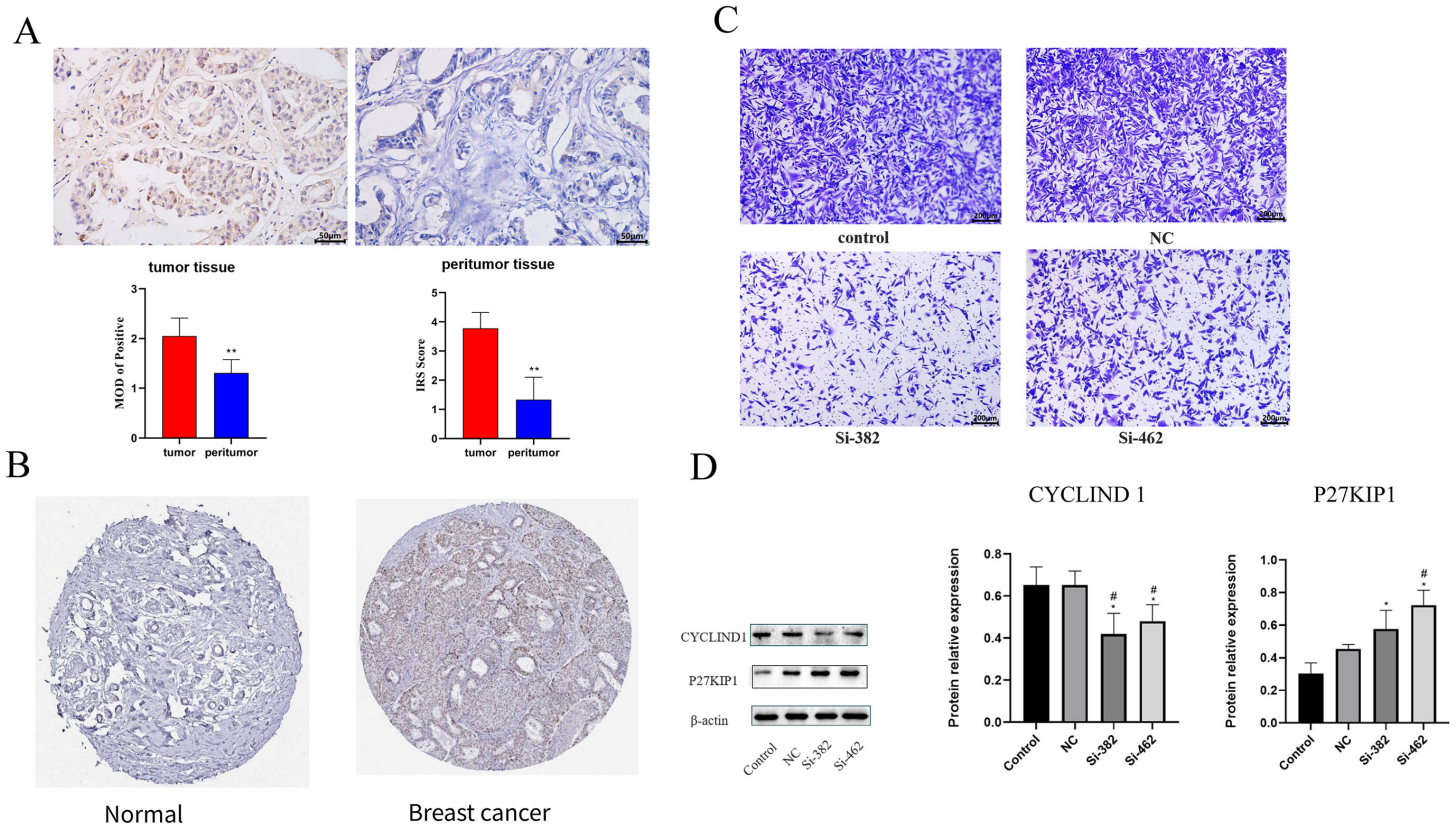
Expression of HMGB3 **(A)** Expression of HMGB3 detected by IHC (**P < 0.01). **(B)** Expression of HMGB3 by HPA. **(C)** Transwell experiment quantifying the number of invasive cells in T24 cells after knockdown. **(D)** Western blot analysis of CYCLIND1, P27KIP1 protein expression levels in cells among various groups after knockdown. (Compared to the control group *P < 0.05, Compared to the NC group ^#^*P* < 0.05).

Furthermore, we examined the expression profiles of HMGB3 in clinical samples from BRCA patients by IHC (**Figure 7A**) analysis and found that consistent with the HPA (**Figure 7B**), HMGB3 expression was significantly increased in BRCA tissues relative to adjacent normal tissues. (*p* < 0.05). The IHC results for antibodies in the six clinical cases are shown in **Supplementary Table S4**. Download the breast cancer dataset GSE42568 from the GEO database, which contains 104 breast cancer samples and 17 adjacent normal tissue samples, retrieve the HMGB3 expression information from the data. HMGB3 expression was found to be significantly higher in breast cancer tissue according to the results. The results are now presented as **Supplementary Figure 5**. Correlation analysis between HMGB3 expression and receptor status (ER, PR, HER2, EGFR, P53, Ki67, CDK2, mTOR2, CDH2, PKMYT1), we analyzed the relationship between HMGB3 expression levels and their status using the GEPIA dataset. The results are now presented as **Supplementary Figure 6** and **7**. A Transwell assay was used to determine how HMGB3 affects the invasive ability of BRCA cells by examining the impact of HMGB3 knockdown on cell invasion. Results indicated that HMGB3 knockdown significantly reduced cell invasion (**Figure 7C**). Using Western blotting to measure the expression levels of cell cycle-associated proteins, specifically G1/S-specific cyclin D1 and the cyclin kinase inhibitor P27KIP1, following the knockdown of HMGB3. In the si-382 and si-462 transfection groups, P27KIP1 protein levels were notably higher, whereas CyclinD1 protein levels were significantly lower compared to the normal control and interference blank groups (**Figure 7D**).

## 4 Discussion

**Figure 8:**
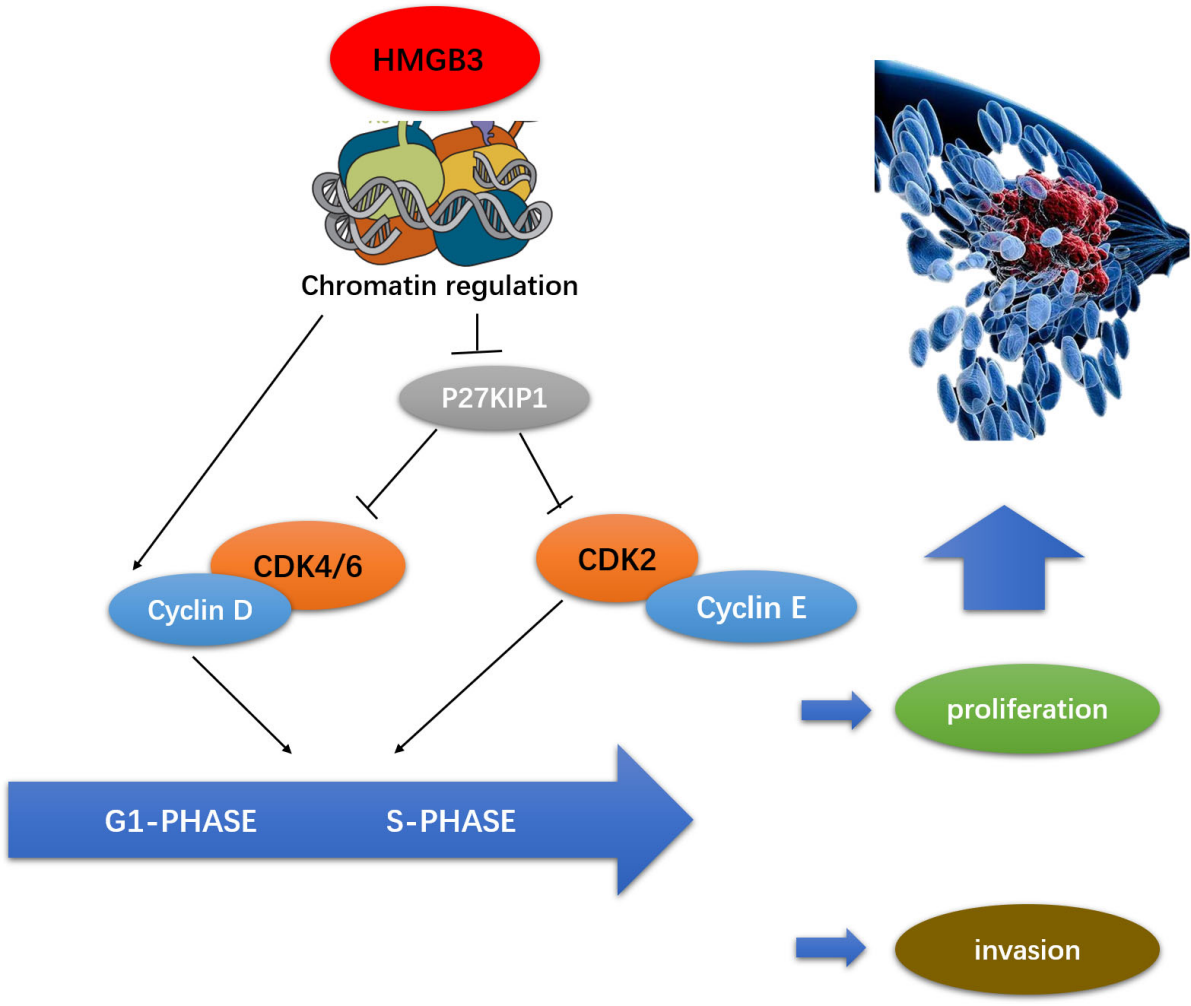
HMGB3 enhances the growth and invasion of breast cancer cells by upregulating Cyclin D1 and downregulating P27KIP1.

Chemotherapy that targets specific pathways continues to be the standard treatment for BRCA, but drug resistance greatly reduces treatment effectiveness. Therefore, it is crucial to continue exploring and identifying new targets. In this study, we found that HMGB3 was significantly elevated in BRCA tissues and associated with cell cycle pathway. Reducing HMGB3 levels hinders the invasion of BRCA cells. We demonstrated that HMGB3 levels were increased in BRCA and contributed to cancer progression. HMGB3 was able to facilitate the entry of cells into next phase through the promotion of the expression of CyclinD1 and the inhibition of the expression of P27KIP1. In essence, this study demonstrated that HMGB3 is a novel regulator of tumor progression in BRCA (**Figure 8**).

BRCA is the most common cancer diagnosed in women and ranks as the second leading cause of death among women globally (17, 18). In 2022, global statistics show that there were 2.3 million new BRCA cases, making up 25% of all female cancer cases, and 670,000 deaths, which accounted for 15.5% of all female cancer fatalities (2). Therefore, if potential biomarkers of BRCA are detected at an early stage and their relationship with patient treatment and prognosis is explored, it is crucial to alleviate patient suffering and improve survival rates. A key indicator of cancer development is the alteration of epigenetics. CRs are indispensable upstream regulators of epigenetics (5). There is a rising body of evidence suggesting that CRs are important for predicting the prognosis of cancer patients. By interfering with the interactions between transcription factors and chromatin-binding regions, MOZ and Menin-MLL chromatin regulatory complexes aid in the proliferation of gastrointestinal stromal tumor cells (19). Evidence suggests that novel CRs can determine the prognosis of colorectal cancer, which is crucial for its immunotherapy (20). It remains to be studied whether CRs have an impact on the prognosis of BRCA patients.

GO and KEGG analysis revealed that pathways such as histone modification, cell cycle, and chromatin remodeling were primarily enriched with differentially expressed CRs. The results indicate that CRs with differential expression might be linked to cell growth in BRCA. Previous studies have demonstrated that histone modifications and chromatin remodeling can alter DNA methylation activity by adding or removing histone modification marks during DNA methylation or by modifying chromatin states. This process influences genomic methylation and promotes cancer development.

It is worth noting that HMGA1, acting as a CR, is significantly elevated in bladder cancer. Downregulation of HMGA1 attenuates proliferation and invasion of bladder cancer cells by modulating the miRNA-221/TP53INP1/p-ERK axis, activates autophagy, and thereby influences the initiation and progression of bladder cancer (10). DNMT1 regulates the stemness and tumorigenicity of hepatocellular carcinoma by mediating BEX1 methylation as a member of the DNA methyltransferase family (11).

ASCL1 serves as a significant regulator of neuroendocrine differentiation. Evidence suggests that ASCL1 aids in the progression of lung adenocarcinoma by modulating cell-autonomous signaling pathways, chemokines, and immune responses (21). During mitosis, as an essential protein for chromosome segregation, ERCC6L supports the proliferation, migration, and invasion of BRCA cells by activating the PLK/CDC25C/CDK1/CYCLIN B and p53/p21/CDK1/CYCLIN B pathways, hastening the progression of the cell cycle (22). A significant association exists between IKZF3 amplification and poor outcomes in HER2-positive BRCA, suggesting its potential as a therapeutic target (23). By reducing H3K36me3, KDM4B suppresses PHGDH, impacting the proliferation, migration, epithelial-mesenchymal transition (EMT), and stemness of BRCA cells (24). PRDF1 and RIZ1 homology domain containing is a subfamily of Krüppel-like zincfingerprotein (ZNF/ZFP). The Gaia family, with its 19 members, plays a role in managing key processes, including the regulation of gene expression and the onset of cancer (25). Studies in the past have used a model that includes six CR-related gene markers, including PRDM11, to successfully forecast BRCA prognosis and sensitivity to immunotherapy (26). This implies that PRDM11 may play a positive role in BRCA outcomes and management. PRDM12, induced by retinoic acid, shows antiproliferative effects by controlling the cell cycle in P19 embryonic carcinoma cells (27). RAD54B, a component of the SW12/SNF2 helicase superfamily, contributes to the reorganization of protein-double-stranded DNA complexes, thereby promoting chromosomal accessibility (28). RAD54B, a component of the SW12/SNF2 helicase superfamily, contributes to the reorganization of protein-double-stranded DNA complexes, thereby promoting chromosomal accessibility (29). The expression of RAD54B has a significant connection to the prognosis of luminal A BRCA (30). SIRT7 belongs to the SIRT family of NAD+-dependent protein deacetylases and serves as a key mediator in various cellular activities (31). SIRT7 and LAP2α collaborate to regulate chromosomal instability and metastasis in BRCA; inhibiting this interaction can prevent the metastasis of BRCA (32). TFF1, a cysteine-rich protein from the tricuspid factor family, is generally expressed in BRCA cells under the control of estrogen (33). By reducing miR-504 levels, TFF1 enhances TP53 protein expression and its transcriptional activity, impacting the onset and progression of gastric cancer (34).

Immunotherapy has recently emerged as a novel cancer treatment approach, capturing the attention of medical researchers worldwide. Research conducted by our group previously has demonstrated the critical importance of digestive system cancers in the tumor immune microenvironment (35). This study aimed to predict the association between the prognostic models of CRs and the immune microenvironment to better grasp their advantages in BRCA during immunotherapy. In the prognostic model, CD8+ T cells are found in the low-risk group. By activating signaling pathways inside tumor cells, CD8+ T cells cause the death of these malignant cells, highlighting their importance as antitumor cells (36). Tumor-associated macrophages, a key component of tumor-infiltrating immune cells, are primarily categorized into M1 and M2 types. By presenting tumor-associated antigens to T cells and producing immunostimulatory factors, M1 macrophages stimulate the proliferation of T cells and NK cells, thereby increasing their antitumor activity (37). By secreting cytokines and growth factors, macrophages with the M2 phenotype can aid in tumor growth, invasion, metastasis, and angiogenesis (38, 39). Previous investigations have shown that the tumor immune microenvironment affects how monocytes differentiate into macrophages (40).

Further analysis of the link between prognostic models and immune checkpoint genes uncovered significant variations across nearly every immune checkpoint. It was also observed that patients in the low-risk category exhibited high expression of immune checkpoint-related genes, suggesting they could benefit more from immune checkpoint inhibitor (ICIs)treatments. Research demonstrates that ICIs provoke immune responses in a range of cancers and are effective in combating cancer (41). Through the STAT3 signaling pathway, PGRN promotes M2 macrophage polarization and PD-L1 expression, aiding breast tumors in immune escape via PD-1/PD-L1 interaction (42), still, the results vary depending on the person. It was revealed through correlation analysis that a higher tumor mutational burden (TMB) was present in the group with elevated risk scores. Studies conducted previously have indicated that TMB is a predictor of cancer patients’ sensitivity to immune checkpoint inhibitors (43). To some degree, this model can discern differences in immunotherapy, assisting in personalized precision treatment.

In this investigation, we found nine CRs (ASCL1, ERCC6L, HMGB3, IKZF3, KDM4B, PRDM11, RAD54B, SIRT7, TFF1) that are co-expressed in both BRCA transcriptome data and scRNA-seq data through parallel analysis, HMGB3 emerged as the most significant gene in BRCA prognosis according to differential expression and survival analysis of nine genes. Subsequently, we authenticated the expression and biological functions of HMGB3 via tissue and cell experiments in BRCA. The IHC analysis showed that HMGB3 expression was significantly higher in BRCA tissue than in the nearby non-cancerous tissue. The Transwell assay revealed that the metastatic capacity of BRCA cells was significantly diminished in the HMGB3 knockdown group, this points to HMGB3’s ability to support the multiplication and invasion of BRCA cells.

High mobility group box (HMGB3) was originally identified as a marker of expression during the development of embryos (44). It has been demonstrated in recent studies that HMGB3 is vital for the processes of DNA replication, recombination, and repair. HMGB3 enhances the growth of cervical cancer cells by modulating the Wnt/β-catenin signaling pathway (45). The suppression of HMGB3 leads to a notable reduction in the migration and proliferation of colorectal cancer cells (46). HMGB3 is influenced by non-coding RNAs and is involved in the development and advancement of diseases. The miR-128-3p/HMGB3 axis is involved in how the downregulation of lncRNA SNHG16 reduces acute lung injury in septic rats (47). HMGB3 was identified as predominantly enriched in BRCA epithelial cells using scRNA-seq data. Evidence indicates that the alteration of epithelial cell polarity, a significant feature of EMT, contributes to the beginning and progression of BRCA (48).

We carried out a single-gene GSEA enrichment analysis to delve deeper into the biological role of HMGB3 in BRCA. The results showed that HMGB3 overexpression is chiefly connected with the cell cycle pathway. Western blot analysis was utilized to examine the connection between HMGB3 and cell cycle proteins (Cyclin D1 and P27KIP1). The results indicated that knocking down HMGB3 caused a reduction in Cyclin D1 protein and an increase in P27KIP1 protein. Research suggests that Cyclin D1 positively influences cell cycle progression, by being overexpressed, it can influence cell cycle activities in BRCA, non-small cell lung cancer, and prostate cancer, thus facilitating cancer progression (49). P27KIP1, which negatively regulates the cell cycle. shows abnormal expression that is closely linked to the initiation and development of cancer (50). These results imply that HMGB3 may enhance cell cycle progression and accelerate the development of BRCA. HMGB3 Contributes to Anti-PD-1 resistance by inhibiting IFN-γ-driven ferroptosis in TNBC (51). Reducing HMGB3 levels can impede cell proliferation *in vitro* and slow down tumor growth in BRCA *in vivo*, with the antitumor effects being mediated by its interaction with HIF1α (52). Thus, HMGB3 can be used as a biomarker for BRCA, offering important insights for the prognostic evaluation of the disease.

Notably, although HMGB3 demonstrated good predictive performance in univariate analysis and in risk scoring models based on CRs, when traditional clinical and pathological factors like age and TNM staging were included in a multivariate regression model, its independent prognostic significance was not statistically significant (p > 0.05). This could be due to the fact that the impact of HMGB3 expression is partly accounted for by traditional clinical variables like TNM staging and tumor grade. There may be an intrinsic connection between its expression levels and the stage of the tumor or the progression of the disease. Indeed, as a cell cycle regulator, increased levels of HMGB3 might play a role in the malignant transformation and progression of tumors, a discovery that is somewhat evident in clinical staging. The results downloaded from the UALCAN database are shown in **Supplementary Figure 8**.

Existing clinical trials and research have validated the reliability of targeted therapies for cancer. Various studies have proposed that CRs abnormalities contribute to the process of tumor development and progression. Our study’s innovation is not in identifying HMGB3 as a gene related to the cell cycle, but in systematically recognizing HMGB3 from the viewpoint of chromatin regulation, its confirmation using parallel scRNA-seq data, its incorporation into a multi-CR prognostic model for breast cancer, and its mechanistic verification in breast cancer cells through the Cyclin D1/P27^KIP1^ pathway. We believe this study offers important supporting evidence for BRCA research through a blend of methodological techniques.

Nonetheless, there are still several limitations that need to be tackled in future studies. Firstly, while our *in vitro* assays provide some initial functional support, the study is lacking *in vivo* validation using animal models, which is vital to confirm HMGB3’s roles in a natural tumor microenvironment. Secondly, even though we applied rigorous batch effect correction, external cohort validation has yet to be introduced. Although we observed stable changes in Cyclin D1 and p27KIP1 levels following HMGB3 knockdown—a finding that has helped us at least partially explain the cause of proliferation, the specific molecular mechanism by which HMGB3 influences these two cell cycle proteins was not further explored by us. Ideally, further experiments like chromatin immunoprecipitation to investigate promoter binding and mRNA stability assays to evaluate post-transcriptional regulation would be required to determine HMGB3’s direct or indirect action. Moreover, further mechanistic research is needed to clarify the regulatory networks linking HMGB3 expression with tumor progression and immune response. Otherwise, we admit that our existing study does not directly demonstrate HMGB3 as a therapeutic target, given that we have not performed *in vivo* experiments or drug-response analyses. Thus, the idea of HMGB3 being a ‘therapeutic target’ should be viewed as initial and hypothesis-generating. Future investigations employing animal models and HMGB3-targeted approaches are necessary to determine the translational potential of HMGB3.

## 5 Conclusion

The prognostic model of BRCA CRs was able to predict the prognosis of BRCA patients; HMGB3 is considered a promising candidate for more in-depth functional and therapeutic studies in breast cancer; HMGB3 promoted the proliferation and invasion of BRCA cells, and HMGB3 was able to facilitate the entry of cells into next phase through the promotion of the expression of CyclinD1 and the inhibition of the expression of P27KIP1.

## Data Availability

The original contributions presented in the study are included in the article/**Supplementary Material**. Further inquiries can be directed to the corresponding author.
