# Supplementary material for: Bulk and single-cell RNA sequencing data identified HMGB3 of chromatin regulators as a breast cancer biomarker associated with the cell cycle
Source: Front Oncol. 2026 Jun 11;16:1835558. doi: 10.3389/fonc.2026.1835558 (PMC13293801; doi:10.3389/fonc.2026.1835558)
Supplement: Supplementary file 1 [file DataSheet1.docx]

**
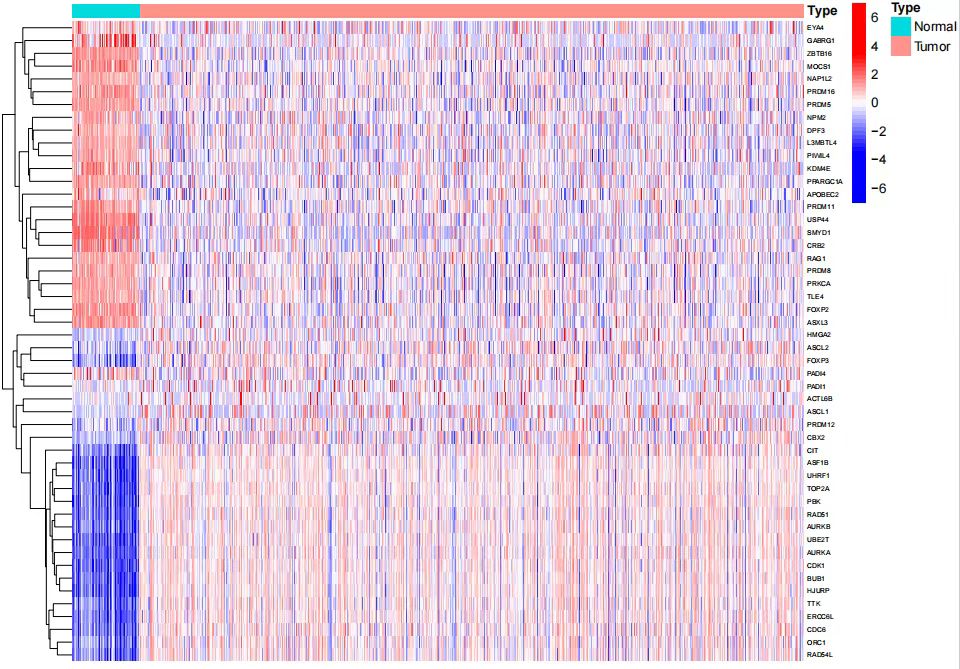
**

**Supplementary Figure1** Heatmap of CRs Expression in BRCA (BRCA, breast cancer).


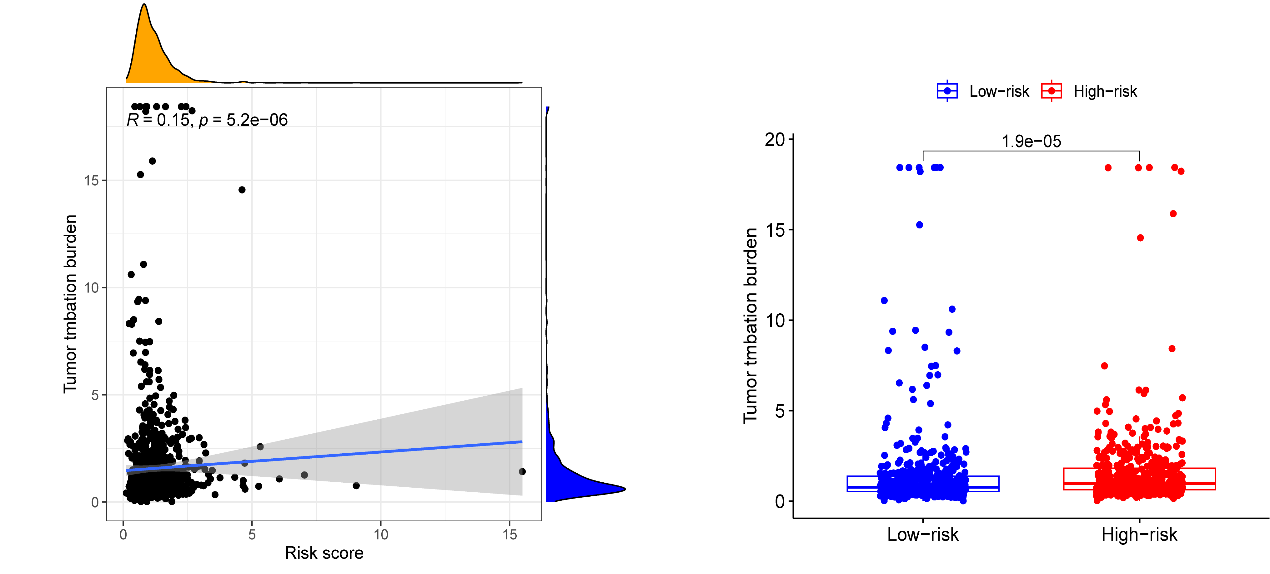


**Supplementary Figure2** TMB of BRCA patients by risk-score (TMB, tumor mutational burden).


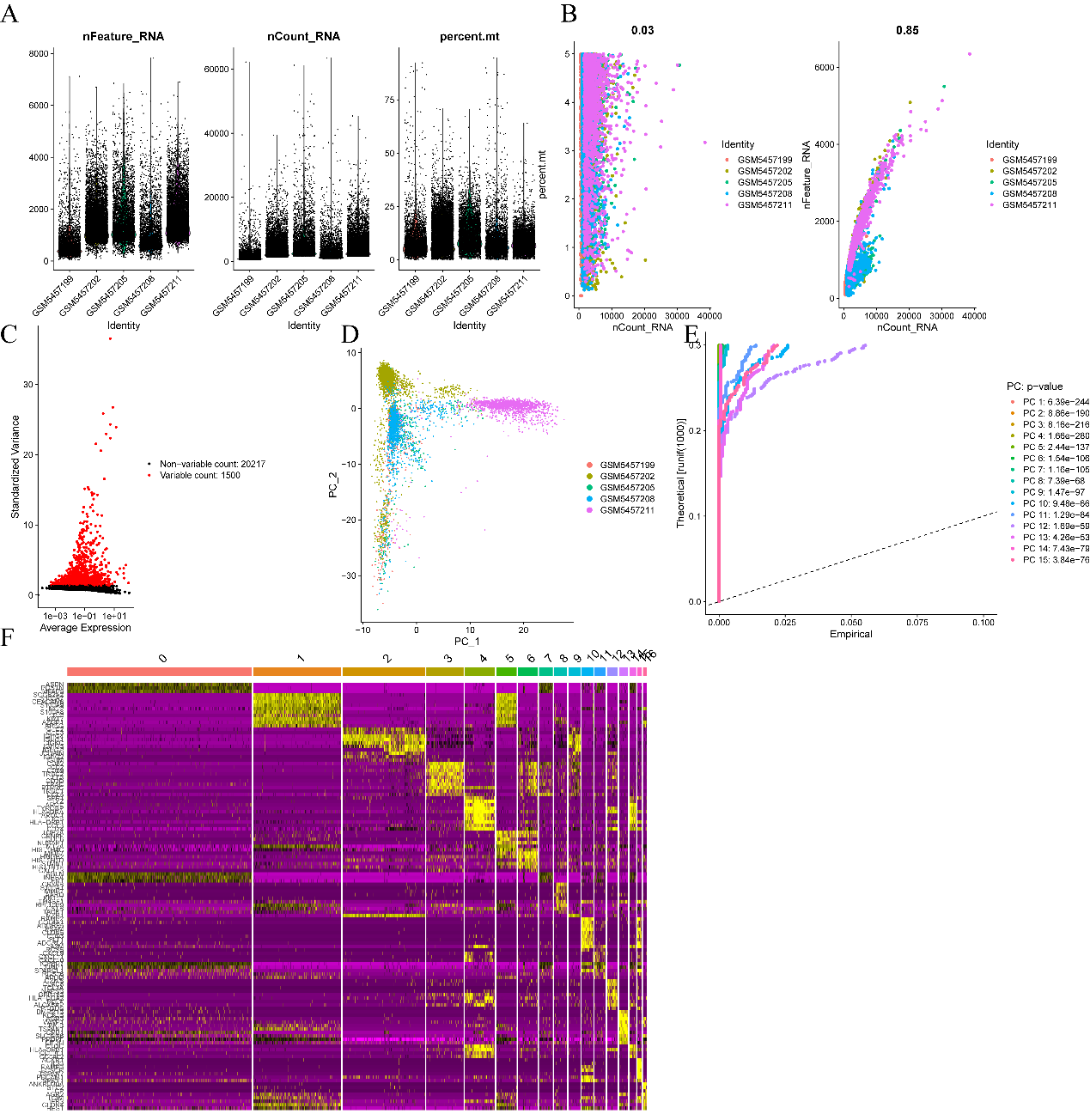


**Supplementary Figure3** (A-C) Quality control of scRNA-seq for five cell sub-

populations;(D, E) The principal component analysis (PCA); (F) constructed between clusters to identify significant marker genes and we exhibited the top 50 in heatmap package (scRNA-seq, single-cell RNA sequencing; PCA, Principal component analysis).

**
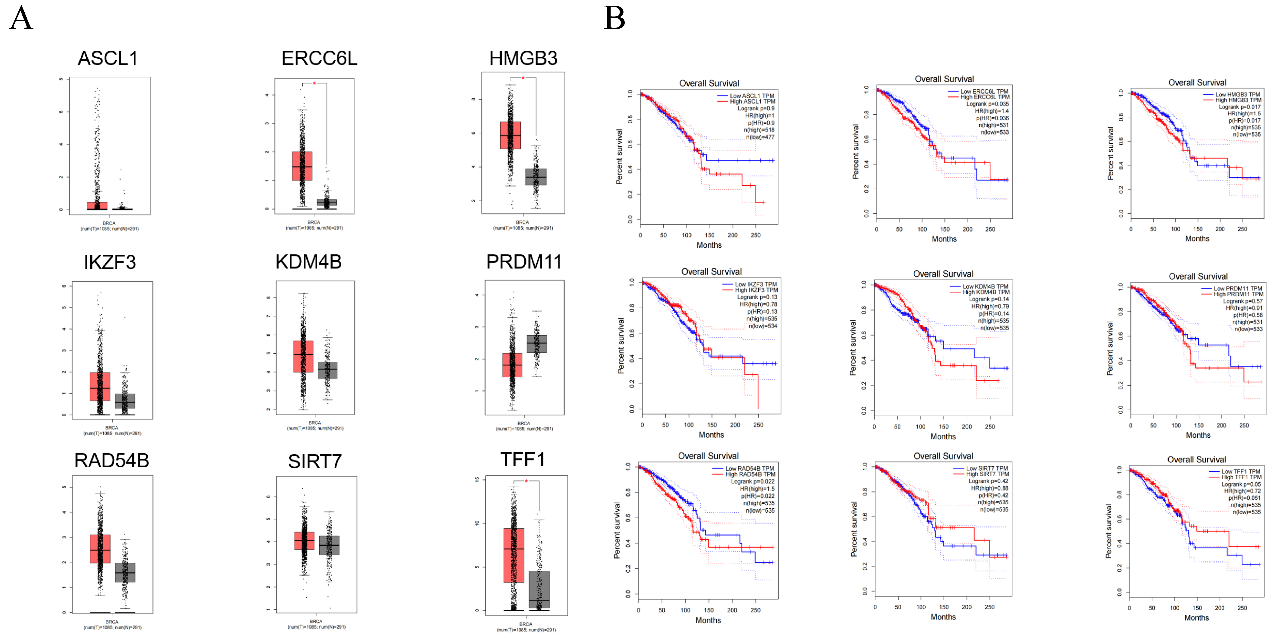
**

**Supplementary Figure4** (A, B) Expression Differences and K-M curves in all common genes in BRCA (K-M, Kaplan-Meier).

**
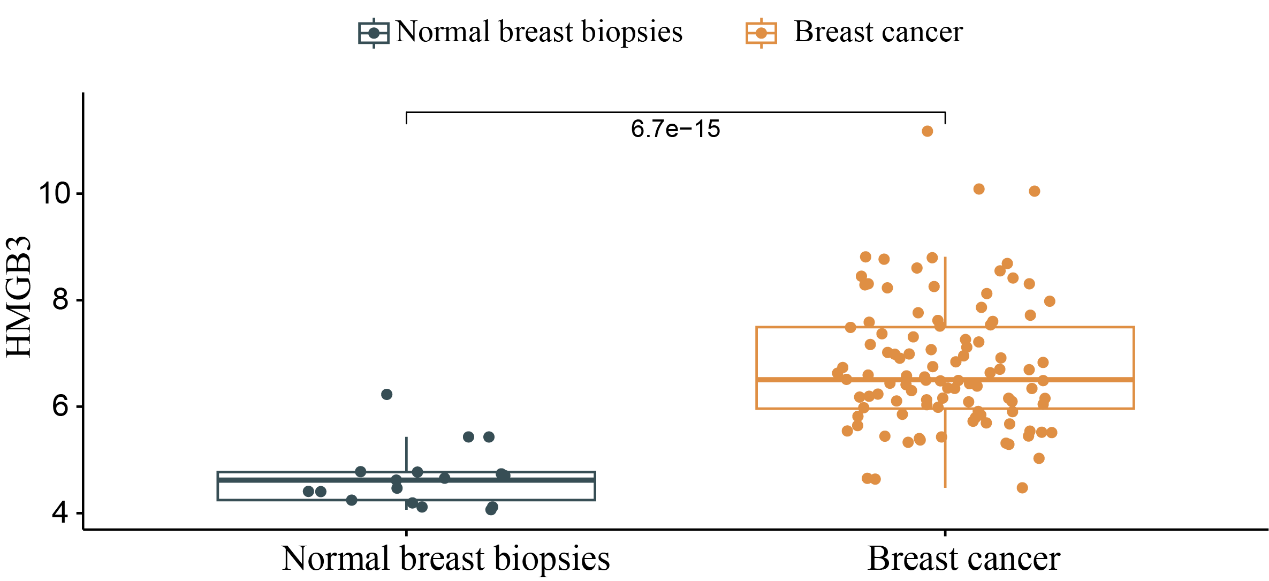
**

**Supplementary Figure 5** Expression of HMGB3 in BRCA from GEO


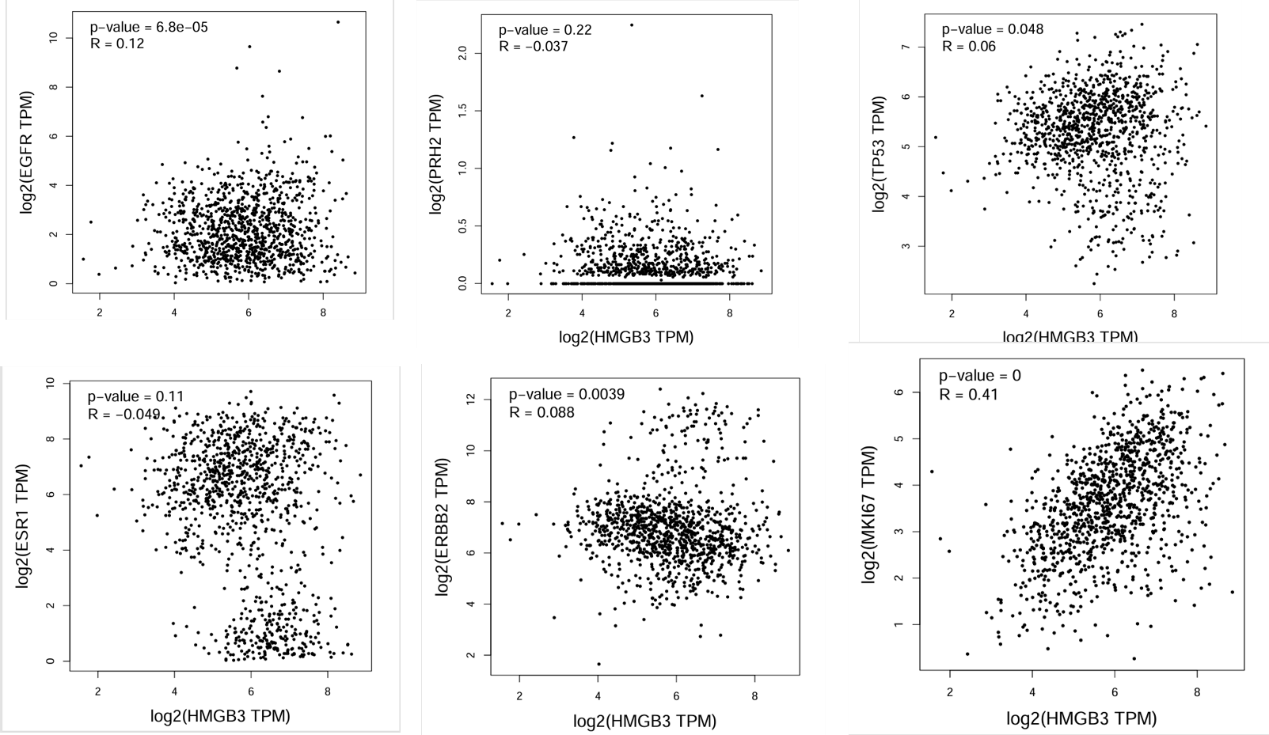


**Supplementary Figure 6** Correlation between HMGB3 and Other Antibodies in BRCA samples from the GEPIA Database (BRCA, breast cancer; GEPIA, Gene Expression Profiling Interactive Analysis).

**
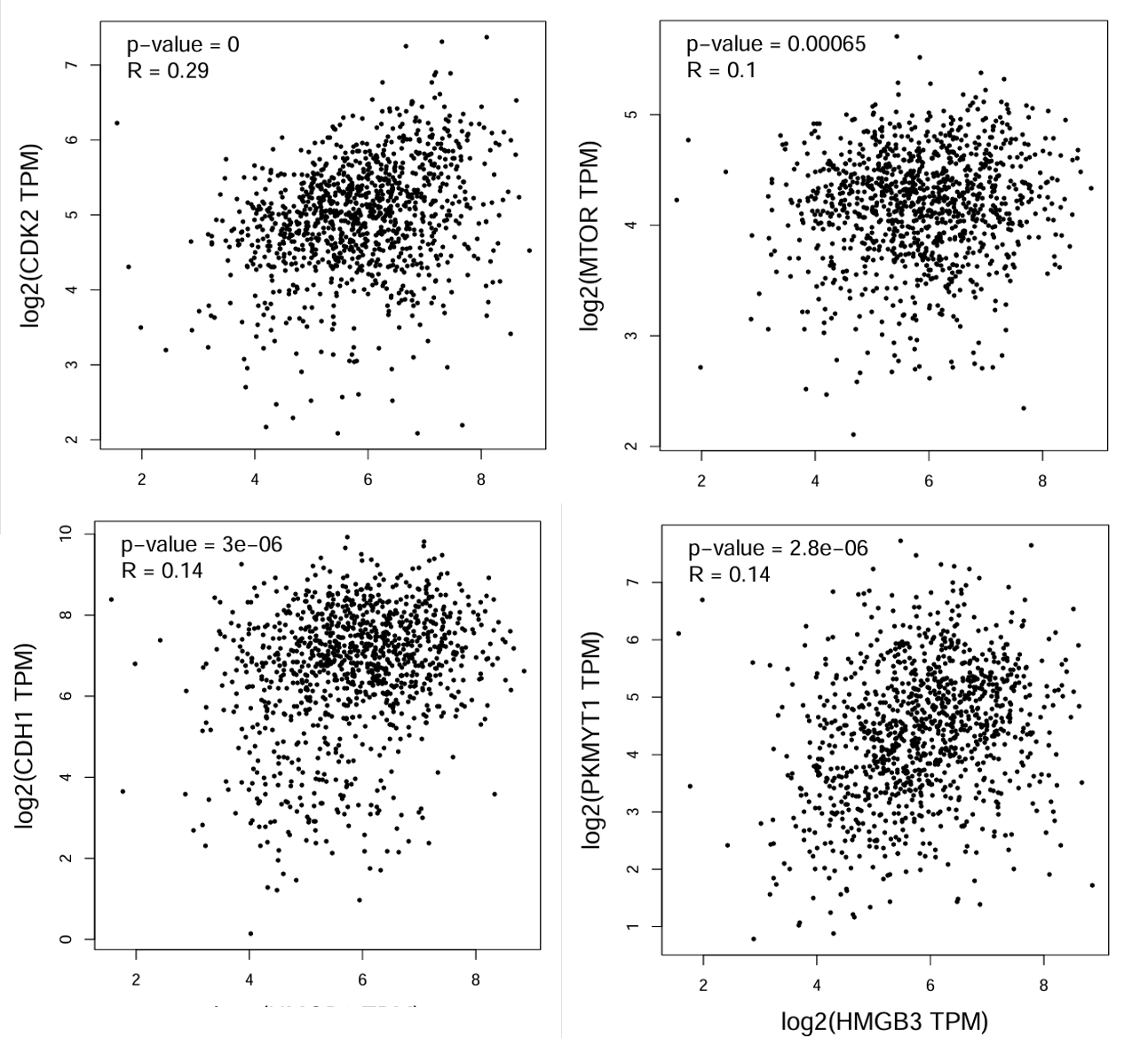
**

**Supplementary Figure 7** Correlation between HMGB3 and Other Antibodies in BRCA Samples from the GEPIA Database (BRCA, breast cancer; GEPIA, Gene Expression Profiling Interactive Analysis).

**
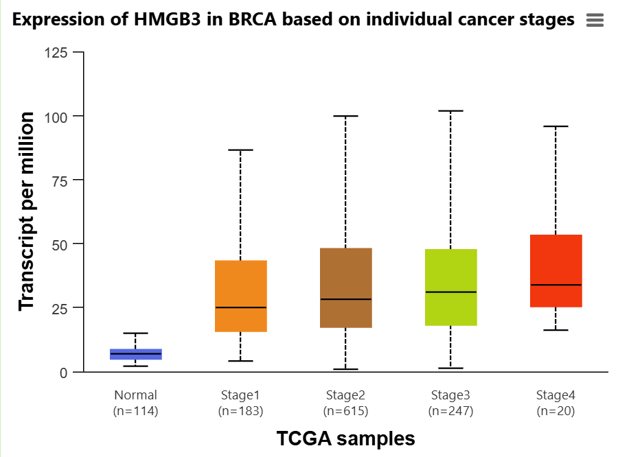
**

**Supplementary Figure 8** Expression of HMGB3 in BRCA based on individual cancer stages from UALCAN database.

**Table1**. Clinical Information of Breast Cancer Transcriptome Data.

| Clinical Information | | Number of sample cases |
| --- | --- | --- |
| age（years） | ＜65 | 645 |
|  | ≥65 | 257 |
| T stage | T1 | 235 |
|  | T2 | 533 |
|  | T3 | 103 |
|  | T4 | 31 |
| N stage | N0 | 447 |
|  | N1 | 298 |
|  | N2 | 102 |
|  | N3 | 55 |
| M stage | M0 | 886 |
|  | M1 | 16 |
| Clinical stage | Ⅰ | 160 |
|  | Ⅱ | 527 |
|  | Ⅲ | 199 |
|  | Ⅳ | 16 |

**Table2**. Antibodies Used and Corresponding Dilutions

| Antibody Name | Dilution rate |
| --- | --- |
| Rabbit Anti P27KIP1 | 1:1000 |
| Mouse Anti CYCLIND1 | 1:5000 |
| HRP conjugated Goat Anti-Mouse IgG (H+L) | 1:2000 |
| HRP conjugated Goat Anti-Rabbit IgG(H+L) | 1:2000 |

**Table3**. Connected Parameters of Genes in the CRs-Informed Prognostic Model for Breast Cancer

| Gene ID | coef | HR | HR.95L | HR.95H | *p*-value |
| --- | --- | --- | --- | --- | --- |
| ASCL1 | 0.120 | 1.127 | 1.002 | 1.268 | 0.047 |
| ERCC6L | 0.056 | 1.058 | 0.751 | 1.490 | 0.747 |
| HMGB3 | 0.047 | 1.049 | 0.875 | 1.257 | 0.608 |
| IKZF3 | -0.319 | 0.727 | 0.598 | 0.884 | 0.001 |
| KDM4B | -0.121 | 0.886 | 0.709 | 1.107 | 0.286 |
| PRDM11 | -0.628 | 0.534 | 0.304 | 0.938 | 0.029 |
| PRDM12 | 1.645 | 5.180 | 0.799 | 33.577 | 0.085 |
| PRDM16 | 0.639 | 1.895 | 1.120 | 3.207 | 0.017 |
| RAD54B | 0.241 | 1.272 | 0.790 | 2.050 | 0.322 |
| SIRT7 | -0.432 | 0.649 | 0.462 | 0.913 | 0.013 |
| TFF1 | -0.049 | 0.952 | 0.902 | 1.005 | 0.077 |

**Table4.** Results from standard antibody IHC procedures in six clinical cases

|  | ER | PR | ERBB2 | EGFR | P53 | Ki67 |
| --- | --- | --- | --- | --- | --- | --- |
| Case1 | +90% | +90% | — | — | + | +70% |
| Case2 | +80% | +85% | — | — | ± | +50% |
| Case3 | +85% | +50% | — | — | + | +60% |
| Case4 | +75% | +50% | ± | — | + | +70% |
| Case5 | +85% | +80% | — | ± | ± | +60% |
| Case6 | +90% | +60% | ±+ | ± | ± | +60% |
